# Semi-automated quantification of vitreal hyperreflective foci in SD-OCT and their relevance in patients with peripheral retinal breaks

**DOI:** 10.1186/s12886-023-03060-7

**Published:** 2023-07-17

**Authors:** P. Strzalkowski, A. K. Schuster, A. Strzalkowska, J. S. Steinberg, S. Dithmar

**Affiliations:** 1 Department of Ophthalmology, Helios HSK Wiesbaden, Wiesbaden, Germany; 2 Department of Ophthalmology, Heinrich-Heine University Duesseldorf, Duesseldorf, Germany; 3 Department of Ophthalmology, University Medical Center of the Johannes Gutenberg, University of Mainz, Mainz, Germany

**Keywords:** Retinal break, SD-OCT, Semi-automated quantification, OCT biomarker, Vitreal hyperreflective foci, Vitreoretinal disorders

## Abstract

**Background:**

Retinal breaks (RB) are emergencies that require treatment to prevent progression of rhegmatogenous retinal detachment. Vitreal hyperreflective foci (VHF) representing migration of RPE cell clusters or interphotoreceptor matrix from the RB are potential biomarkers. The aim of this study is to investigate VHF in RB-patients using SD-OCT.

**Methods:**

The retrospective cross-sectional study included RB patients from our Department of Ophthalmology, HSK Wiesbaden who underwent macular SD-OCT (SPECTRALIS®, Heidelberg Engineering, Germany) on both eyes. VHF, defined and quantified as foci that differ markedly in size and reflectivity from the background speckle pattern, were assessed for presence and frequency. The RB-affected eyes were the study group (G1), the partner eyes the control group (G2).

**Results:**

160 consecutive patients with RB were included. Age was 60 ± 10.2 years (52% female). 89.4% of G1 and 87.5% of G2 were phakic (p = 0.73). 94.4% (n = 151) were symptomatic. Symptom duration was 8.0 ± 10.1 days in G1, 94.4% (n = 151) showed VHF versus 5.6% (p < 0.0001) in G2, of which 75% (n = 6) showed asymptomatic lattice degenerations. Detectable VHF showed a strong association of OR = 320 (95% CI, 110–788, p < 0.0001)) with respect to symptomatic RB. Sensitivity and specificity were 94.7% and 94.7%, respectively.

**Conclusions:**

Most eyes with symptomatic RB show vitreal VHF in SD-OCT. Detected VHF are strongly associated with RB, and our semi-automated greyscale reflectivity analysis indicates that VHF likely originate from photoreceptor complexes torn out of the RB area that migrate into the vitreous cavity. The presence of VHF may indicate RB and should lead to a thorough fundus examination in both symptomatic and asymptomatic cases.

## Background

The detection and treatment of retinal breaks is important as they can lead to retinal detachment. Visualisation of retinal breaks may be challenging due to cataract formation, missing pupillary response to mydriatics, posterior synechiae, anterior capsular phimosis in pseudophakic eyes, corneal edema, glare of IOL or vitreous opacities [1, 2]. Whether retinal defects are detected also depends on the clinical experience of the examiner [3]. SD-OCT has become a mainstay in retinal diagnostics [4] and can assist in the process of detection of vitreal hyperreflective foci (VHF).

When the blood-retinal barrier (BRB) is compromised around the retinal break, RPE cells are exposed to a significant level of cytokines and growth factors in the vitreous cavity. This exposure triggers activation of the RPE cells, leading to their proliferation, initiation of epithelial-mesenchymal transition (EMT) and development of migratory abilities towards the vitreous cavity or intraretinal layers through the retinal break.

Such VHF might represent migrated RPE cell clusters [5] or interphotoreceptor matrix [6] from retinal breaks.

Furthermore, VHF can not only occur in retinal breaks, but can also be observed in other ocular conditions, such as diabetic retinopathy (DRP) [7], uveitis [8–10] and retinal vein occlusion [11]. In DRP, VHF have been found to correlate with the severity and duration of the disease, as well as with the presence of macular edema. Similarly, VHF have been observed in uveitis, where it may be an indicator of inflammatory activity and disease progression [9]. In retinal vein occlusion, VHF have been associated with diabetic macular edema (DME) and poor visual outcomes [11].

Therefore, analyzing the unique characteristics and importance of VHF in each particular disease can provide valuable insights into the underlying biological processes responsible for these conditions.

Understanding the relationship between VHF and retinal breaks might be clinically important as many OCT examinations for macular diagnostics are performed without dilating the pupil [12], as well as improving artificial intelligence (AI) algorithms in ophthalmology [13]. Incorporating VHF as a feature in AI models can enhance their accuracy in detecting peripheral retinal breaks, even when the pupil is not dilated during routine OCT examinations [14, 15]. This could ultimately result in better patient outcomes by enabling earlier detection and treatment of retinal breaks and preventing vision loss [16].

We investigated the characteristics and relevance of VHF using high-resolution SD-OCT imaging in patients with confirmed peripheral retinal breaks and analyzed the association with presence of retinal breaks.

## Methods

### Clinical data collection

In this cross-sectional study, electronic health records of all emergency patients between 01/2021 and 01/2022 presented with peripheral retinal breaks without retinal detachment at the Department of Ophthalmology (n = 181), Helios HSK Wiesbaden, Germany, were retrospectively analysed. The respective healthy partner eyes served as a control group, thus it reflects an intra-individual study design.

Both eyes had been examined by retinal specialists in mydriasis with a Goldmann 3-mirror contact lens.

The following parameters for both eyes from each patient were extracted: gender, age, laser treatment, best-corrected visual acuity (BCVA) in logMAR, refraction, intraocular pressure (IOP), lens status (phakic or pseudophakic), onset of symptoms (eye floaters/photopsia) in days, number of retinal breaks, location of retinal breaks, central retinal thickness in SD-OCT, history of ocular trauma and previous ophthalmic surgeries.

### Inclusion criteria

We included consecutively all patients with a confirmed peripheral retinal break and a routinely performed SD-OCT of the macular region (n = 160) of both eyes.

### Exclusion criteria

Patients without high-resolution OCT (n = 20), uveitis (n = 1) or other diseases like proliferative diabetic retinopathy, retinal vascular occlusion, previous complications during cataract surgery and ocular trauma (n = 0) were excluded. All consecutive patients, regardless of previous retinal treatments, were included in the description of the study population, however for the association analysis, we excluded the corresponding pair of eyes if there was previous retinal laser treatment or vitreoretinal surgery in the control group (n = 9).

### Optical coherence tomography

**Fig. 1:**
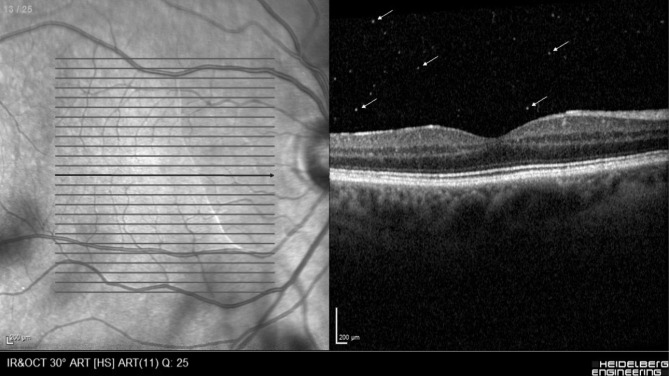
Standard macular OCT scan of a right eye with infrared image (left side) and B-scan (right side). VHF are exemplarily marked with white arrows on the B-scan

All SD-OCT images (high-speed mode, 20° × 20° (6.2 × 6.2 mm), ART minimum 9 frames averaged, 25 B-scan, distance between scans 257 μm) were obtained routinely by an experienced operator using SPECTRALIS® (Heidelberg Engineering, Heidelberg, Germany). Macular OCT images were centred on the foveal depression. All 25 OCT B-scans of each eye were analysed individually, and the scan with the greatest amount of observable VHF was included in the study. VHF were graded on OCT scans defined as foci in the vitreous cavity, which were clearly distinguishable from the background speckle pattern in size and reflectivity [17]. The intra-observer repeatability of VHF detection was investigated on 80 randomly selected OCT scans. The percent overall agreement was 98.8% κ = 0.98 (95% CI, 0.93-1.0). The severity of VHF was graded as **VHF 0** = absent, **VHF 1** = mild (VHF < 4), **VHF 2** = moderate (4 ≥ VHF < 15), or **VHF 3** = severe (15 ≥ VHF) [18] (Fig. 1).

### Qualitative OCT scan analysis

**Fig. 2:**
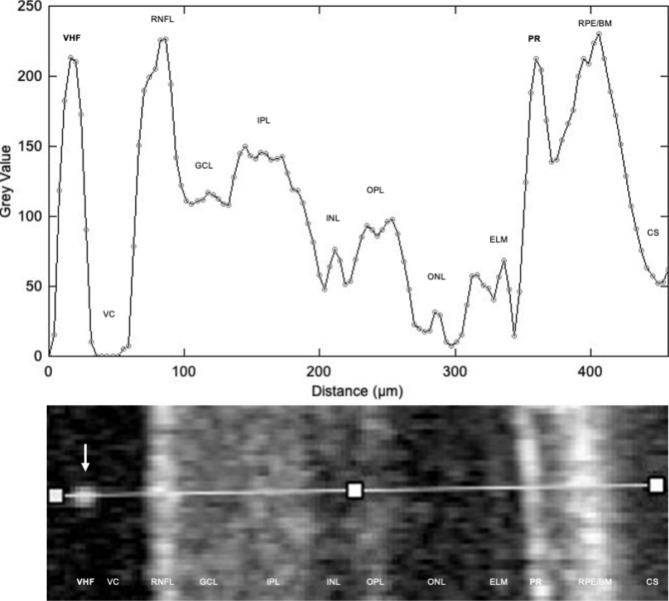
Measurement of grey values with annotation of vitreal hyperreflective foci (VHF), vitreous cavity (VC), retinal nerve fiber layer (RNFL), ganglion cell layer (GCL), inner plexiform layer (IPL), inner nuclear layer (INL), outer plexiform layer (OPL), outer nuclear layer (ONL), external limiting membrane (ELM), photoreceptor layer (PR), retinal pigment epithelium/Bruch membrane complex (RPE/BM) and choroidal stroma (CS) and the corresponding ROI in an 8-bit greyscale OCT scan. The VHF is indicated by the arrow in the OCT scan

1. Raw OCT scan sets from both eyes of all patients were exported from Heidelberg Eye Explorer (HEYEX Version 2.5.5, Heidelberg Engineering, Germany). Images were imported into ImageJ based FIJI open-source image processing software (https://imagej.net/software/fiji/) [19] and converted into 8-bit greyscale. The brightness is expressed in 256 levels from black = 0 to white = 255. As our region of interest (ROI) we set a straight line on a macular B-scan orthogonal to the retinal surface through a single VHF (reference point), vitreous cavity (VC), retinal nerve fiber layer (RNFL), ganglion cell layer (GCL), inner plexiform layer (IPL), inner nuclear layer (INL), outer plexiform layer (OPL), outer nuclear layer (ONL), external limiting membrane (ELM), photoreceptor layer (PR), retinal pigment epithelium/Bruch membrane complex (RPE/BM) and choroidal stroma (CS) (Fig. 2). The grey value of the ROI was expressed as a value between 0 and 255.

2. For the particle analysis and calculation of the VHF area and diameter the measurement scale was then set according to the 200-micrometre scale specified on the OCT scan. In order to differentiate VHF from the background speckle, the threshold for each OCT scan was adjusted so that the background noise disappeared and only VHF remained. After thresholding, a watershed function was used to segment possible overlapping VHF to allow accurate identification of all individual particles. The ROI was set to analyse only the OCT vitreous window. The circularity index was set to 0.8-1.0. The Analyze Particles menu in ImageJ was used to obtain particle counts and size. Particle size analysis was carried out using an automated method.

### Statistical analysis

Statistical analysis was performed using GraphPad Prism9 (GraphPad Software, San Diego, USA) for Mac. For statistical analysis, BCVA was converted in logarithm of the minimum angle of resolution (logMAR) scale. Categorical variables were presented as absolute and relative frequencies, whereas mean and standard deviation were computed for approximately normal-distributed continuous variables, otherwise median and interquartile range. Evaluation of data normality was performed using the Shapiro-Wilk test. Categorical variables were compared using Fisher’s exact test. For calculation of difference in greyscale we used ANOVA with Dunnett’s correction for multiple comparisons. We calculated odds ratio (OR) and 95% CI by Fisher’s exact test using the Baptista-Pike method. Spearman correlation for ordinal data was used. Non-normally distributed continuous variables were compared using Mann-Whitney and Wilcoxon tests. For multiple comparisons, non-parametric Kruskal-Wallis test and post hoc Dunn’s test were used. All statistical tests were two-sided and p-value < 0.05 was considered statistically significant.

### Ethical considerations

Written informed consent and IRB approval for analysing anonymized data in this study was waived in concordance with national and local legislation by the Ethics Committee of the Medical Chamber Hessen (Germany) and *Helios Health Insititue* (HHI), because of the monocentric, retrospective nature of the study.

The study is reported using the *STrengthening the Reporting of OBservational studies in Epidemiology* (STROBE) statement [20] and performed in accordance with the ethical standards set forth in the 1964 Declaration of Helsinki and the Health Insurance Portability and Accountability Act.

## Results

### Patients’ characteristics

181 consecutive patients presented with a peripheral retinal break between 01/2021 and 01/2022 at the Department of Ophthalmology, HSK Wiesbaden. 160 patients, who received a routine macular SD-OCT examination, were included in this study. Demographic data is shown in Table 1.

**Table 1:** Demographic data of study cohort -

Demographic data of study cohort			
	Entire study group		
Male/female n, (%)	77 (48.1) / 83 (51.9)		
Age (years) mean ± SD	60 ± 10.2		
Duration of symptoms (days) mean ± SD	8.0 ± 10.1		
	**Eyes with retinal breaks (G1)**	**Fellow eyes (G2)**	*p*-value
Number of eyes (n)	160	160	1
Symptomatic patients (n)	151	0	< 0.0001
BCVA (logMAR) mean ± SD	0.11 ± 0.18	0.11 ± 0.27	0.02*
Refraction (dpt) mean ± SD	-1.2 ± 3.0	-1.18 ± 3.0	0.22*
IOP (mmHg) mean ± SD	16.1 ± 2.7	16.4 ± 2.9	0.06*
Central retinal thickness (µm) mean ± SD	282 ± 22.2	289 ± 42.3	0.01**

*Wilcoxon matched-pairs signed rank test, **Paired t-test

The mean age was 62 ± 9.6 years for males and 59 ± 10 years for females, respectively (p = 0.08). There was a statistical significant difference in BCVA between the study eyes (0.1 ± 0.18) and the control eyes (0.1 ± 0.27) (p = 0.02). The refraction (dpt) was − 1.2 ± 3 in the study eyes and − 1.18 ± 3 in the control eyes (p = 0.22). Central retinal thickness as measured with SD-OCT was slightly thinner in study eyes (282 ± 22.2 μm) than in control eyes (289 ± 42.3 μm; p = 0.01). 89.4% study eyes (n = 143) and 87.5% of control eyes (n = 140) were phakic (p = 0.73).

### Duration of symptoms

Of the 160 patients, 151 patients (94.4%) reported symptoms (mouches volantes/photopsia), while 9 patients (5.6%) were asymptomatic in the study group. Mean duration of symptoms in the study group was 8.0 ± 10.1 days. There was no statistically significant difference in durations of symptoms between males (7.1 ± 8.6 days) and females (8.3 ± 11 days) (p = 0.86). The mean duration of symptoms until first presentation at the emergency department of our eye clinic was 10.4 ± 12.1 days for the VHF 1 group, 5.5 ± 6.5 days and 5.0 ± 3.8 days for the VHF 2 and VHF 3 groups, respectively. This difference was statistically significant (p = 0.02). None of the asymptomatic patients in the study group (n = 9) showed VHF in OCT (VHF 0).

### Number and localisation of retinal breaks

In study eyes, a single retinal break was identified in 86.9% (n = 139), two retinal breaks were found in 12.5% (n = 20) of eyes and three retinal breaks in 0.6% (n = 1). A cumulative number of 182 retinal breaks were detected in the study group. Of these, 96.2% (n = 175) were horseshoe breaks, while only 4% (n = 7) represented round holes. Most retinal breaks (75.8%, n = 138) were located in the upper hemisphere. The most common clock hour was 12 o’clock (13.2%, n = 24) followed by 11 o’clock (12.1%, n = 22), 10 o’clock (11.5%, n = 21), 9 o’clock (11.5%, n = 21), 1 o’clock (10.4%, n = 19) and 2 o’clock (10.4%, n = 19). The most frequent inferior localisation of a retinal break was 6 o’clock (11.5%, n = 21).

In the control eyes, 5.6% (n = 9) had a retinal break, of which one patient had a retinal detachment as an incidental finding at initial presentation. In 3.8% (n = 6), lattice degeneration areas could be identified.

### Frequency of VHF in the study and control group

While in the study group 94.4% of eyes (n = 151) showed VHF, in control eyes VHF were seen in 10.7% (n = 17) only.

In study eyes, the frequency of VHF 0, VHF 1, VHF 2 and VHF 3 was 9, 70, 56 and 25 eyes, respectively. All study eyes without visible VHF in SD-OCT (n = 9) were asymptomatic.

In the control fellow eyes, the frequency of VHF 0, VHF 1 and VHF 2 was 143, 16 and 1 eye, respectively. VHF 3 could not be detected in any fellow control eye. Of the 17 patients with visible VHF in the control eye, 52.9% (n = 9) had prior treatment for retinal breaks or retinal detachment by laser coagulation, cryocoagulation or vitreoretinal surgery. In the remaining 47.1% patients (n = 8), lattice degeneration areas were identified in 75% (n = 6). The one patient with VHF2 in the fellow eye was diagnosed with an asymptomatic peripheral retinal detachment at the time of initial presentation.

### Characteristics of VHF

#### Area and diameter of VHF

We measured 125 single VHF in 20 OCT scans from 20 randomly selected patients. Mean area and diameter of these VHF were 123.1 ± 97.3 µm^2^ and 35.1 ± 16.0 μm, respectively. The diameter of VHF was below 20 μm in 25.6% (n = 32), between 20 and 30 μm in 17.6% (n = 22), between 30 and 40 μm in 20% (n = 25) and between 40 and 50 μm in 16% (n = 20).

#### Greyscale intensity of VHF

The analysis of the grey values based on 20 individual VHF measurements in 10 OCT scans from 10 study group patients with VHF 3 resulted in a mean grey value for VHF as a reference point of 198 ± 11.4 compared to VC (1.38 ± 0.9), RNFL (218 ± 14.8), GCL (129 ± 12.7), IPL (163 ± 10.7), INL (83.4 ± 12.1), OPL (102 ± 9.5), ONL (61.6 ± 11.1), ELM (108 ± 9.6), PR (200 ± 11.3), RPE/BM (238 ± 11.6) and CS (52.8 ± 9.9).

**Fig. 3:**
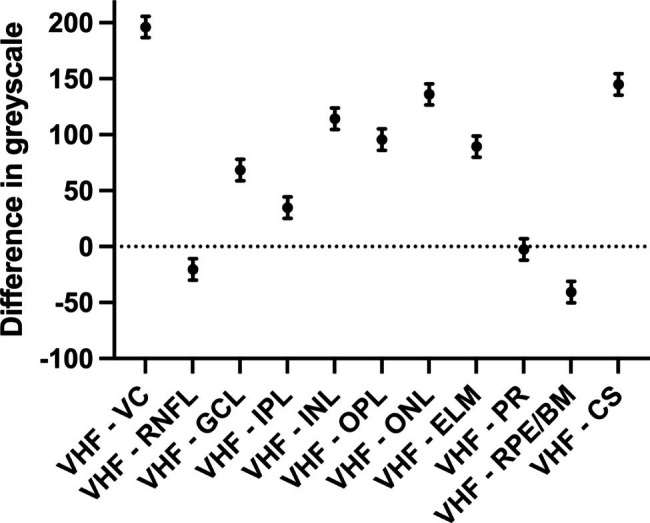
Difference in mean greyscale values and standard deviation between the reference point vitreal hyperreflective foci (VHF), vitreous cavity (VC), retinal nerve fiber layer (RNFL), ganglion cell layer (GCL), inner plexiform layer (IPL), inner nuclear layer (INL), outer plexiform layer (OPL), outer nuclear layer (ONL), external limiting membrane (ELM), photoreceptor layer (PR), retinal pigment epithelium/Bruch membrane complex (RPE/BM) and choroidal stroma (CS) (n = 20)

The mean greyscale difference was 196 (95% CI, 187–206) for VHF vs. VC, -20.3 (95% CI, -29.9 to -10.7) for VHF vs. RNFL, 68.3 (95% CI, 58.7–77.9) for VHF vs. GCL, 34.7 (95% CI, 25.2–44.3) for VHF vs. IPL, 114 (95% CI, 105–124) for VHF vs. INL, 95.7 (95% CI, 86.1–105) for VHF vs. OPL, 136 (95% CI, 126–146) for VHF vs. ONL, 89.4 (95% CI, 79.8–98.9) for VHF vs. ELM, -40.7 (95% CI, -50.3 to -31.1) for VHF vs. RPE/BM, -2.5 (95% CI, -12.1-7.1) for VHF vs. PR and 145 (95% CI, 135–154) for VHF vs. CS. The mean difference of the grey values differed significantly from all retinal structures (p < 0.001) except for the PR (p = 0.99, ANOVA with Dunnett’s correction for multiple comparisons) (Fig. 3).

### Refraction versus VHF frequency in study group

**Fig. 4:**
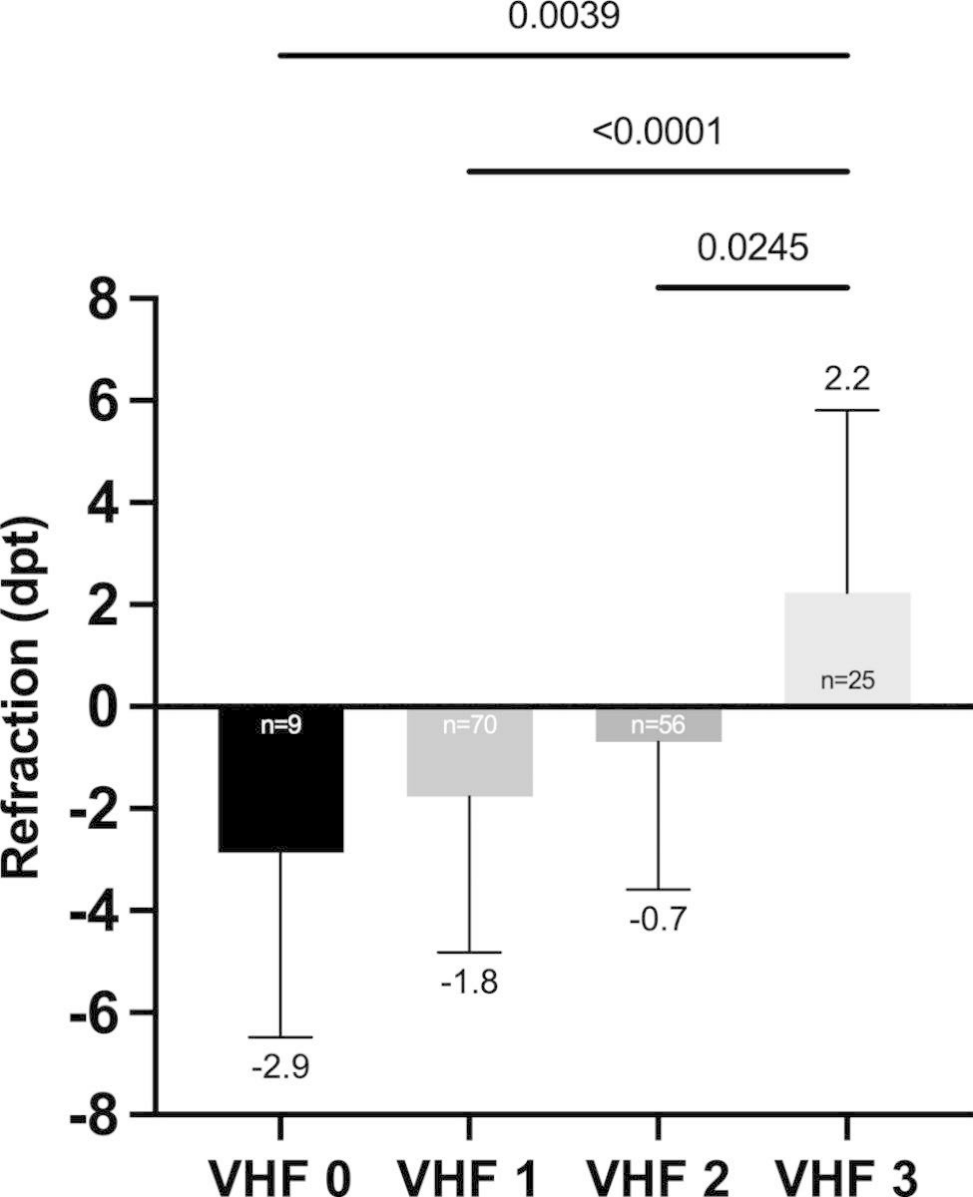
Comparison of refraction (dpt) in relation to VHF frequency

**Fig. 5:**
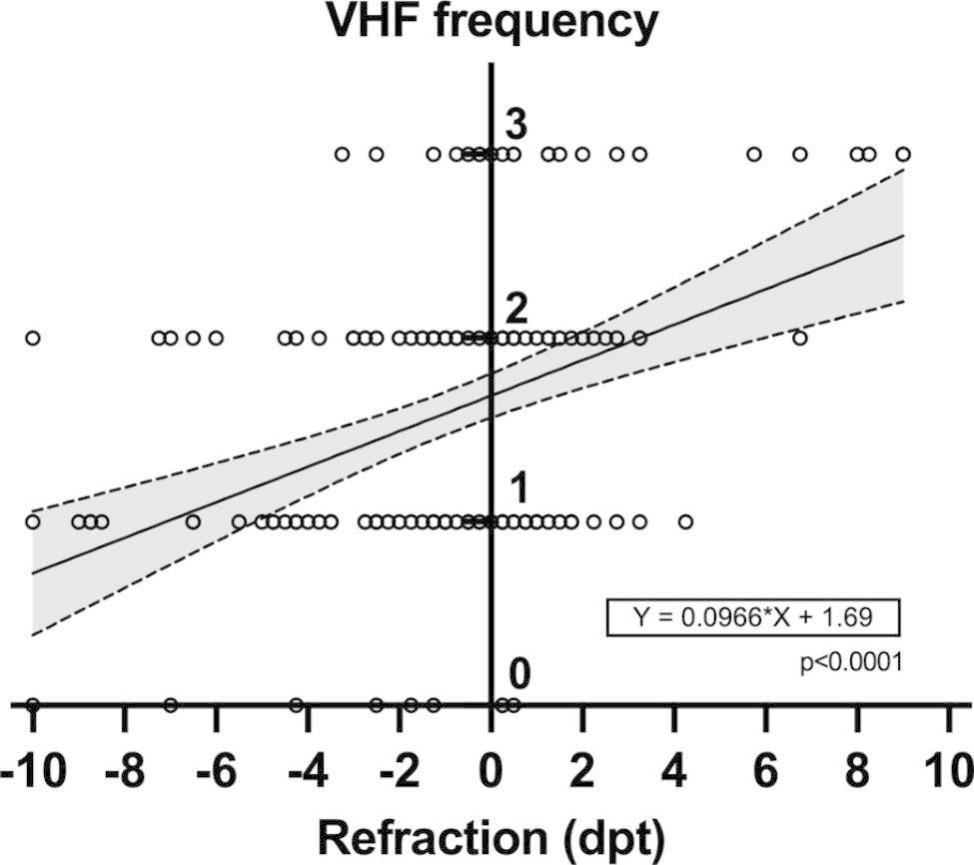
Correlation between refraction (dpt) and VHF frequency

The refraction showed a significant difference between VHF 3 (+ 2.2 dpt) vs. VHF 0 (-2.9 dpt) (p = 0.004), VHF 3 vs. VHF 1 (-1.8 dpt) (p < 0.0001) and VHF 3 vs. VHF 2 (-0.7 dpt) (p = 0.02) (Fig. 4). There was a positive correlation between the frequency of VHF and refraction (*ρ* = 0.37; p < 0.0001) (Fig. 5).

### Association analysis

We performed an association analysis between VHF and retinal breaks in our study sample using intraindividual control eyes in a cohort of symptomatic retinal break patients. There was an association of OR = 141 (95% CI, 60.3–306, p < 0.0001) for this relation.

In addition, we excluded a total of 9 pairs of eyes due to pre-treated retinal breaks or retinal detachment in the fellow eye. Then, 94.7% (n = 143) in the study eyes and 5.3% (n = 8) in the fellow eyes showed VHF. There was an association between retinal breaks and VHF (OR = 320; 95% CI, 110–788, p < 0.0001).

## Discussion

The aim of this study was to investigate the characteristics and relevance of VHF using high-resolution SD-OCT imaging in patients with confirmed peripheral retinal breaks. In the present study, visible VHF in SD-OCT showed a strong association OR = 320 (95% CI, 110–788, p < 0.0001) with respect to retinal breaks. The sensitivity and specificity were 94.7% and 94.7%, respectively.

Furthermore, in the SD-OCT based greyscale analysis of the VHF compared to the vitreous cavity, retinal layers and choroidal stroma, we were able to show for the first time that the origin of VHF were probably photoreceptor complexes which were torn out of the retinal break area and migrated into the vitreous cavity. In addition, refraction showed a positive linear correlation with VHF frequency. Myopic patients had less VHF compared to hyperopic patients.

As early as the 1920s, Jules Gonin described the pathogenesis of rhegmatogenous retinal detachment (RRD) [21]. A full thickness retinal break in combination with a vitreoretinal traction leads to accumulation of subretinal fluid and separation of neurosensory retina (NSR) from retinal pigment epithelium (RPE) [22]. Considering that the global prevalence of people with myopia will rise significantly until 2050 [23] and the fact that myopia is one of the most important risk factors for retinal breaks [24], they are and will remain one of the ophthalmologic emergencies that must be diagnosed and treated early to prevent progression into RRD, which can lead to visual impairment or even visual loss particularly in macular involvement [25, 26].

The presence of pigmented cells in the anterior vitreous cavity (known as Shafer’s sign) is almost pathognomonic for retinal breaks [27], although other studies have shown a wider range of incidence between 83% [28] and 92% [29].

Qureshi et al. investigated the inter-observer reproducibility of Shafer’s sign based on the examiner’s clinical experience in 47 patients. The authors not only found examiner agreement dropped considerably from 78.2 to 61.7% with less experience, but also that a retinal break was only present in 63.8% [30]. Since the recognition of Shafer’s sign de-pends particularly on the experience of the examiner, it cannot be completely relied upon for clinical assessment.

OCT examination has become a fundamental diagnostic tool in many retinal diseases due to the rapid technical progress since the 1990s [30, 31] and is characterised by high repeatability, reproducibility, reliability and examiner-independence [31, 32].

Oh et al. studied patients with acute symptomatic posterior vitreous detachment (PVD) who had VHF detected by OCT and found a positive correlation between frequency of VHF and probability of retinal breaks [18]. In the here presented large cohort of patients we confirm this observation and provide a simple examination protocol that can be used to assess the risk of retinal breaks using standard OCT examination.

We found no association between VHF severity graded as number of VHF and the number of retinal breaks. Furthermore, we observed a positive correlation between VHF frequency and refraction. On the one hand, this could be explained by the fact that the distribution pattern of VHF in the vitreous cavity differs between myopic and hyperopic eyes, due to the axial length. Myopic eyes have a higher vitreous cavity volume compared to hyperopic eyes [33] and the same amount of VHF means a lower concentration in myopic eyes, assuming that the VHF are equally distributed in the vitreous cavity.

On the other hand, it is conceivable that the detectability and the distribution pattern of the VHF in the standard OCT scan may change after a posterior vitreous detachment [34].

We were able to demonstrate for the first time that VHF show no differences to the photoreceptor layer in terms of grey values (p < 0.001), but significant differences to all other retinal layers in SD-OCT. This supports the thesis that VHF originate from the photoreceptor layer. The origin of visible VHF in OCT has not yet been fully elucidated. Adhesion between the NSR and RPE is determined in particular by the interphotoreceptor matrix (IPM). In RD due to retinal breaks, this fragile adhesion homeostasis is compromised [35]. Vitreous aspirate analysis of patients with retinal detachment (RD) found a correlation between Shafer sign and interphotoreceptor complexes rather than intact RPE cells [35]. An experimental study in Yucatan micropigs inhibited the synthesis of chondroitin sulfate proteoglycan, the main component of IPM, by intravitreal injection of p-nitrophenyl-beta-D-xylopyranoside (xyloside). Only treated animals developed RD, emphasizing the importance of IPM in adhesion forces between NSR and RPE [36]. Consistent with these study results, our greyscale reflectivity analysis of VHF may suggest that the origin could most likely be photoreceptor complexes.

How long VHF remain in the vitreous cavity is not clear yet. Oh et al. performed OCT follow-up in 14 patients after 2.8 ± 1.5 weeks and found a decrease of VHF [18]. In our study, although we did not do any follow-up, we found a correlation between symptom duration (in days) and VHF frequency in the study group. While patients in the VHF 3 and VHF 2 group reported symptoms for about 5 days, patients in the VHF 1 group experienced symptoms for 10 days until they presented to our eye clinic. All patients in the VHF 0 group were asymptomatic.

This may indicate that the OCT detectable VHF frequency may change over time since the onset of symptoms.

Our study has several limitations. VHF measured approximately 35.1 μm on average. Therefore, it is possible that not all VHF could not be detected due to the distance of 257 μm between each OCT scan. In further studies, the OCT device settings should be increased in terms of scan density, length and depth of the vitreous window. This study is a cross-sectional study, thus we cannot state on cause of VHF. A further prospective case-control study is planned to investigate the likelihood of a retinal break in the case of VHF as underlying pathology. As we only included patients with confirmed peripheral retinal break in this study, we cannot state on other diseases, such as uveitis, which may also lead to VHF [9, 17] in the vitreous body. In addition, patients were examined only at the initial emergency presentation, thus no follow-up examination was conducted.

## Conclusion

Visible VHF in macular SD-OCT were associated with peripheral retinal breaks in this intraindividual cross-sectional study. VHF may serve as a predictive factor for alterations in the vitreoretinal interface, as not only patients with retinal breaks more often showed VHF, but also patients with asymptomatic lattice degenerations more frequently had bilateral VHF. Our SD-OCT-based semi-automated greyscale reflectivity analysis provides for the first-time evidence supporting the theory that the origin of VHF is most likely from photoreceptor complexes torn out of the retinal break area that have migrated into the vitreous cavity. Furthermore, a positive linear correlation was found between the frequency of VHF and refraction, with hyperopic patients having a higher frequency of VHF than myopic patients.

This strong relationship may also be important because in clinical practice many OCT examinations for macular diagnosis are performed without pupil dilation.

Understanding the strong relationship between VHF and retinal breaks is crucial for clinical diagnosis, especially since many OCT exams for macular diagnostics are performed without pupil dilation. Paying attention to VHF in the vitreous cavity may contribute to early detection of retinal breaks and prevention of retinal detachment. Moreover, incorporating VHF as a feature in AI models can enhance their accuracy in detecting retinal breaks, enabling earlier diagnosis and treatment for better patient outcomes.

## Data Availability

The datasets used and/or analysed during the current study are available from the corresponding author on reasonable request.

## Abbreviations

BCVA: best-corrected visual acuity
BRB: blood-retinal barrier
CI: confidence interval
CS: choroidal stroma
ELM: external limiting membrane
EMT: epithelial-mesenchymal transition
GCL: ganglion cell layer
INL: inner nuclear layer
IPL: inner plexiform layer
IOP: intraocular pressure
logMAR: logarithm of the minimum angle of resolution
ONL: outer nuclear layer
OPL: outer plexiform layer
OR: odds ratio
PRE/BM: retinal pigment epithelium/Bruch membrane complex
PR: photoreceptor layer
RB: retinal break
RNFL: retinal nerve fiber layer
ROI: region of interest
RRD: rhegmatogenous retinal detachment
SD: standard deviation
SD-OCT: Spectral-domain optical coherence tomography
VC: vitreous cavity
VHF: vitreal hyperreflective foci
